# Low-Dose Radiotherapy Has No Harmful Effects on Key Cells of Healthy Non-Inflamed Joints

**DOI:** 10.3390/ijms19103197

**Published:** 2018-10-16

**Authors:** Lisa Deloch, Michael Rückert, Rainer Fietkau, Benjamin Frey, Udo S. Gaipl

**Affiliations:** Department of Radiation Oncology, Universitätsklinikum Erlangen, Friedrich-Alexander-Universität Erlangen-Nürnberg (FAU), 91054 Erlangen, Germany; Lisa.Deloch@uk-erlangen.de (L.D.); Michael.Rueckert@uk-erlangen.de (M.R.); Rainer.Fietkau@uk-erlangen.de (R.F.); Benjamin.Frey@uk-erlangen.de (B.F.)

**Keywords:** low-dose radiotherapy, fibroblast-like synoviocytes, osteoclasts, osteoblasts, immune cells, joint

## Abstract

Low-dose radiotherapy (LD-RT) for benign inflammatory and/or bone destructive diseases has been used long. Therefore, mechanistic investigations on cells being present in joints are mostly made in an inflammatory setting. This raises the question whether similar effects of LD-RT are also seen in healthy tissue and thus might cause possible harmful effects. We performed examinations on the functionality and phenotype of key cells within the joint, namely on fibroblast-like synoviocytes (FLS), osteoclasts and osteoblasts, as well as on immune cells. Low doses of ionizing radiation showed only a minor impact on cytokine release by healthy FLS as well as on molecules involved in cartilage and bone destruction and had no significant impact on cell death and migration properties. The bone resorbing abilities of healthy osteoclasts was slightly reduced following LD-RT and a positive impact on bone formation of healthy osteoblasts was observed after in particular exposure to 0.5 Gray (Gy). Cell death rates of bone-marrow cells were only marginally increased and immune cell composition of the bone marrow showed a slight shift from CD8^+^ to CD4^+^ T cell subsets. Taken together, our results indicate that LD-RT with particularly a single dose of 0.5 Gy has no harmful effects on cells of healthy joints.

## 1. Introduction

Radiotherapy (RT) is a vital part of multimodal cancer therapies and approx. 60% of all tumor patients receive it during their treatment [1]. However, next to the induction of cell death and acute inflammation, RT is also applied in low and intermediate doses, the so called low-dose radiotherapy (LD-RT), and thereby exerts analgesic and anti-inflammatory effects [2]. These beneficial effects have been observed in the clinic for a long time: Sokolow, for example, experienced a reduction of pain levels in arthritic children that were treated with X-rays, shortly after the discovery of X-rays in 1895 [3]. This lead to frequent usage of LD-RT and the description of favorable effects, especially in degenerative diseases of the spine and joints starting in the 1920’s. In the 1970’s, X-rays were even considered to be the most effective physical-therapeutic approach in this kind of diseases. Later on, however, concerns about the safety of LD-RT arose and treatment numbers decreased [4].

Today, medical guidelines suggest the usage of LD-RT for degenerative and inflammatory diseases, such as arthroses or heel spurs, where the majority of patients report analgesic effects after therapy [5,6]. LD-RT should be applied with a single dose per fraction of 0.5 Gray (Gy) twice a week with total doses ranging from 3 to 6 Gy [5,7,8], as several clinical studies have already proven that a single dose of 0.5 Gy is as effective as 1.0 Gy/fraction [9,10,11]. Pre-clinical data indicate that 0.5 Gy ameliorates inflammatory conditions even better than 1.0 Gy and discontinuous-dose dependencies are regularly observed [12]. Other indications for LD-RT are hyperproliferative processes, such as benign fibromatous diseases [5,8,13,14].

Nevertheless, the restrains against this form of therapy are mostly owed to the fear of radiation-induced side effects, even though no significant increase in cancer incidents has been observed. This lack of evidence is either owned to the fact that there is no increased risk of carcinogenesis after LD-RT or because the existing risks are too delicate to be portrayed [15,16]. In general, radiation-related risks are considered to be very small, and calculations of the theoretical risk of LD-RT amounts to 20–40 per million treated patients, meaning that it is below the spontaneous formation rate of malignancies by four orders of magnitude [6]. Still, especially in younger patients, risks and benefits need to be carefully evaluated and patients should be aware of the possible side effects of LD-RT, no matter how small they are [9,14].

During LD-RT, patients receive only a fraction (5–10%) of the total doses that tumor patients receive during their therapy [17]. Therefore, it is not surprising that the molecular effects of LD-RT differ from those of high dose RT. Even though the anti-inflammatory properties become increasingly well examined, there is still a lack in the understanding of its functional and molecular mode of action. Among the known effects of LD-RT is a reduced adhesion capacity of peripheral blood mononuclear cells (PBMCs) to endothelial cells that were stimulated with Interleukin (IL)-1β in a dose range from 0.1 to 0.5 Gy [18]. Macrophages also show anti-inflammatory properties after being exposed to LD-RT [19,20]. In addition to these in vitro studies, examinations with experimentally induced arthritis in animal models have been carried out. Mice that have received irradiation with 0.1, 0.3, and 0.6 Gy before a lipopolysaccharide challenge showed reduced leukocyte adherence with increased TGF-β secretion confirming the in vitro results [21]. In a carrageenan air pouch model of acute inflammation, mice received doses of up to 5 Gy of X-rays. While numbers of inflammatory cells remained largely unaffected, cytokine levels as e.g., of TNF-α varied with radiation dose and time point. In addition, the experimenters observed the attenuation of iNOS expression after LD-RT, concluding that ionizing radiation is able to functionally modulate inflammatory cells [22]. A patient study in painful musculoskeletal diseases that examined the effects of radon spa therapy, another form of LD-RT treatment, showed decreased pain perception in patients alongside an overall anti-inflammatory immune response and decreased bone resorption [23,24,25].

It has to be stressed that all of the aforementioned studies were carried out in an inflammatory setting. In order to better understand the molecular mechanisms and also the possible risk factors of LD-RT, it is important to additionally perform analyses in a healthy environment. We therefore examined for the first time under healthy conditions the effects of low and intermediate as well as one high dose (2.0 Gy) of irradiation on cell types that play a major role in disease development and upkeep in rheumatoid arthritis (RA). We hereby focused on fibroblast-like synoviocytes (FLS), osteoclasts, and osteoblast.

FLS play a key role in the preservation of the rheumatic lesion [26,27]: they are cells of the mesenchymal lineage that show many characteristic features of fibroblasts; however, they display an altered morphology and activation pattern in comparison to fibroblasts [27,28]. They show a distinctive, anchorage-independent growth pattern, can develop a resistance to contact inhibition, and show aggressive behavior with increased invasiveness alongside mediation of inflammation [28,29]. Next to their own aggressive behavior, they also produce an abundance of cytokines and proteases that are also involved in cartilage destruction and the upkeep of inflammation [27].

In addition to inflammation, RA is characterized by a progressive destruction of bone and cartilage, with osteoclasts, as the bone-resorbing cells, being found abundantly at the site of arthritic lesions [30]. Several studies have identified osteoclasts as the main responsible cell type for bone damage in RA [31,32,33]. Under normal healthy conditions, osteoclastogenesis and bone resorption are kept in balance with the formation of bone matrix and regulation of osteoclast numbers and activity. Factors that are released by resorbing osteoclasts or the bone matrix couples bone resorption and build-up. However, in RA, bone homeostasis is misbalanced [34].

Correspondingly, osteoblast function seems to be compromised in RA, as it has also been shown for other diseases affecting the bone, such as in osteoporosis and osteoarthritis (OA) [35]. Furthermore, an excess of pro-inflammatory cytokines also works in favor of bone destruction rather than protection [36]. This might also explain why pathogenic bone erosion is often found at sites of inflammation. Many cell types that are involved in the upkeep of inflammation are also able to express receptor activator of nuklear factor κ B (NFκB) ligand (RANK-L) [37]. RANK-L is, together with macrophage colony-stimulating factor (M-CSF), one of the two essential factors for osteoclastogenesis [38]. Its counterpart is osteoprotegerin (OPG), which is a molecule acting as a negative regulator for osteoclastogenesis. Thus, the RANK-L/OPG ratio can be used as an indicator for bone resorption/built up.

As all these cells, FLS, osteoclasts, and osteoblasts, exist within close proximity within the synovial joint and are affected by each other, we focused our analyses on the impact of LD-RT on factors of these cells that have the potential to either stimulate or abrogate inflammation and/or joint destruction. Additionally, we took a closer look on the functionality of these cells in order to gain insights on how exposure to low or intermediate doses of radiation affects cells in healthy joints. Since knowledge on the osteoimmunological properties of radiation in dependence of the initial inflammatory state is scarce, investigations on healthy, non-inflamed cells are therefore urgently needed. A deeper understanding of these processes will not only help to better understand the cellular and molecular mechanisms of LD-RT, but also help to assess a useful risk-benefit consideration of LD-RT treatments.

## 2. Results

### 2.1. LD-RT Has a Slight Impact on FLS Cell Growth and Apoptosis but Not Invasiveness

In RA, FLS are characterized as a hyperproliferative cell type with a certain resistance to apoptosis [39,40]. We therefore characterized them with regards to cell growth and cell death rates after a treatment with various doses of X-rays. It has also been shown that FLS are able to aggressively infiltrate healthy tissue [41], thus, we aimed to investigate whether LD-RT is able to reduce invasiveness and migration of FLS through scratch and matrigel invasion assays.

**Figure 1:**
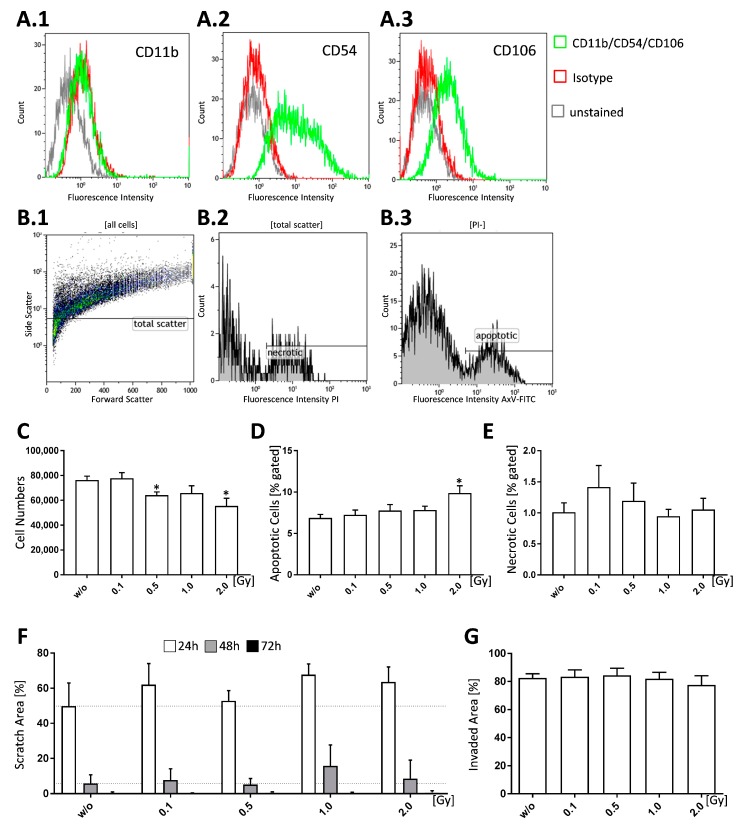
Low doses of ionizing radiation have no significant impact on cell death and migration properties of FLS cultures of healthy mice. Healthy FLS cultures obtained from C57Bl/6 mice were phenotyped at passage 5, seeded and analyzed 96 h after the treatment with low, intermediate, and high doses of ionizing radiation. Cell pools that were tested via flow cytometry were considered to be FLS when being CD11b^−^ (**A.1**), CD54^+^ (**A.2**), and CD106^+^ (**A.3**). **B.1** to **B.3** shows gating strategy for cell death analysis of FLS via flow cytometry. Cell death was determined after staining of the cells (**B.1**., total scatter) with AxV-FITC/PI (**B.2**, **B.3**). Vital cells were defined as AxV^−^/PI^−^, necrotic ones as PI^+^ (**B.2**, **E**), and apoptotic cells as AxV^+^, but PI^−^ (**B.3**, **D**). Cell numbers (**C**) of living FLS were counted using a Neubauer chamber. Cell migration and invasiveness was tested via scratch assay (**F**) and matrigel invasion assay (**G**). For the scratch assay, cells were irradiated after a scratch was applied; pictures were taken every 24 h and scratch area was quantified using Tscratch (V1.0, Copyright T.Geback, M.Schulz, Zurich, Switzerland). The initial scratch area was set to 100% and scratch area of the consecutive days was calculated accordingly. For assessment of invasiveness cells were seeded in a matrigel coated transwell, irradiated and incubated for 3 days. Cells were then stained with crystal violet and invaded area was assessed using ImageJ. Depicted is data from one exemplary phenotyping experiment (**A.1**–**3**). Data for cell growth (**C**), cell death (**D**,**E**), scratch assay (**F**), and invasion assay (**G**) was examined in three independently performed experiments, each being performed in triplicates, whereas, in the case of scratch assay the three same fields of view were captured every time. Data is presented as mean ± SEM and analysed by two-tailed Mann-Whitney-U test in comparison to untreated controls. (*p < 0.05).

In order to positively identify FLS cultures, cells were tested for the expression of specific surface markers via flow cytometry: FLS express intercellular adhesion molecule 1 (ICAM1, cluster of differentiation (CD) 54) that mediates their interaction with leukocytes [27,28]. Vascular adhesion molecule 1 (VCAM1, CD106) is also found on synoviocytes but is seldomly found on non-intimal lining mesenchymal cells making it a reliable marker for FLS. Both, ICAM1 and VCAM1, bind to integrins; however, ICAM1 expression is not only found in FLS, but also in fibroblasts, while constitutive VCAM1 expression is unique to FLS [27]. Our FLS pools were found to be CD11b^−^, CD54^+^, and CD106^+^ (Figure 1A.1–A.3).

In order to examine proliferative properties, we counted the cell numbers 96 h after irradiation with various doses of X-rays. We found that treatment with 0.5 and 2.0 Gy resulted in significantly reduced cell numbers (*p* = 0.0116 and 0.0111) (Figure 1C) in comparison to untreated controls. In terms of cell death 96 h after irradiation, apoptotic cells showed a significant increase only after irradiation of FLS with 2.0 Gy (*p* = 0.0244) (Figure 1D), while necrotic cells were not significantly altered (Figure 1E). However, cell death rates in general were relatively low. When looking at cell migration (Figure 1F) and cell invasiveness (Figure 1G), we found no significant effects after LD-RT. Cell migration, as visualized by scratch assay (Figure 1F), was reduced after irradiation with 0.1, 1.0 and 2.0 Gy, whereas invasiveness, as tested via matrigel invasion assay (Figure 1G), showed no differences.

### 2.2. LD-RT of 0.5 Gy Induces TGF-β Release and Has a Potential Impact on Molecules Involved in Cartillage and Bone Destruction

As RA FLS produce large amounts of inflammatory cytokines, such as IL-6, we aimed to analyze whether this accounts also for healthy, non-inflamed FLS.

**Figure 2:**
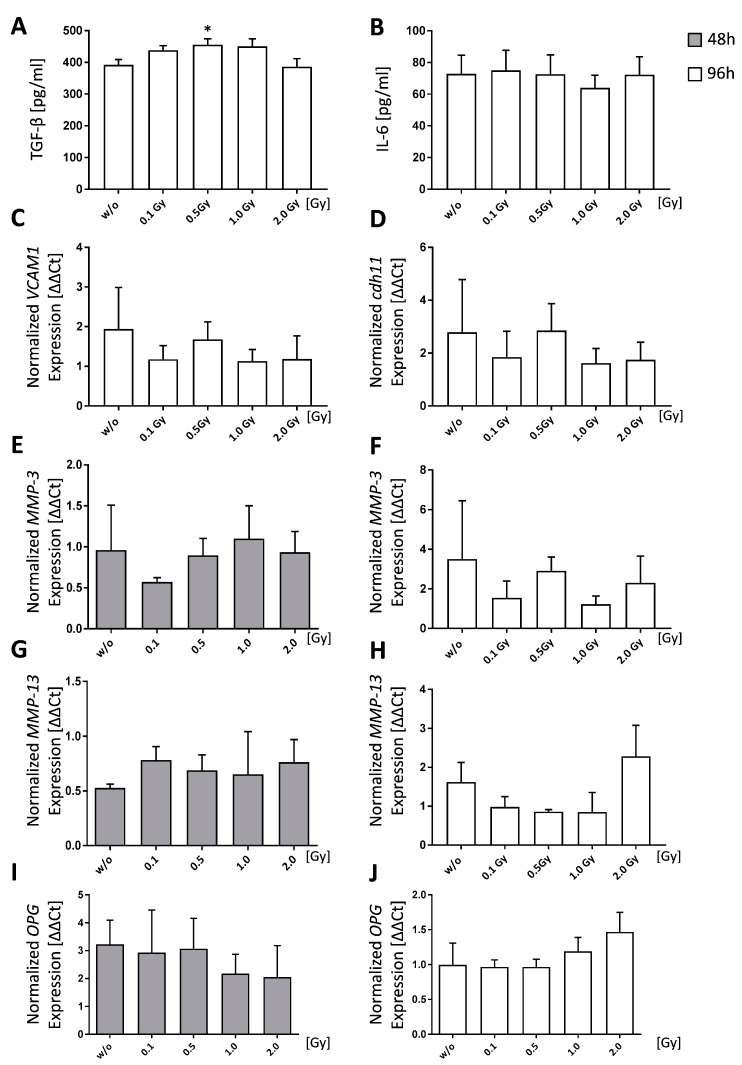
Low doses of ionizing radiation show only a minor impact on cytokine release by healthy fibroblast-like synoviocytes (FLS) as well as on molecules involved in cartilage and bone destruction. Healthy FLS cultures obtained from C57Bl/6 mice were seeded and analyzed 48 h (grey bars) or 96 h (white bars) after the treatment with low, intermediate, and high doses of ionizing radiation. (**A**,**B**) show cytokine levels for TGF-β and IL-6, as determined via ELISA. (**C**–**J**) show quantitative PCR (qPCR) data after phenol-chloroform RNA extraction with subsequent gene expression analysis via SYBR green qPCR analyses. Measurements were normalized using the housekeeper genes *RPS18*, *GAPDH*, and *ACTB*. Shown is the data of three FLS cell pools examined in three independent experiments, each being performed in triplicates. Data is presented as mean ± SEM and analyzed by two-tailed Mann-Whitney-U test in comparison to untreated controls. (* *p* < 0.05).

TGF-β secretion by FLS was measured via ELISA 96 h after LD-RT and a significant increase (*p* = 0.0315) of TGF-β after a single dose of 0.5 Gy was detected (Figure 2A). In contrast, no significant impact of radiation on IL-6 secretion was observed (Figure 2B).

VCAM1, one of the most prominent FLS marker, is an adhesion molecule that mediates leukocyte adhesion to the vascular endothelium [27]. As LD-RT has been shown to reduce leukocyte adhesion [42], we were interested whether it also has an impact on VCAM1 in FLS. Furthermore, FLS have been shown to secrete destructive molecules, such as matrixmetalloproteinases (MMPs) and cadherin-11 (cdh11), which have been shown to be involved in cartilage destruction in rheumatic lesions [43,44,45]. Likewise, FLS are known to stimulate osteocalstogenesis [36,46]. Thus, we aimed to investigate for possible altered gene expression patterns in relevant genes of interest. All data was normalized to the housekeeper genes *ribosomal protein 18S* (*RPS18*), *glyveraldehyde-3-phosphate dehydrogenase* (*GAPDH*), and *β-actin* (*ACTB*).

We found expression levels of *VCAM1* and *cdh11* to be slightly reduced after irradiation with 0.1, 1.0 and 2.0 Gy, whereas no reduction was observed after 0.5 Gy (Figure 2C,D). MMP-3 levels were slightly altered in a time-dependent manner (Figure 2E,F): 48 h after irradiation, *MMP-3* expression was slightly reduced at a dose of 0.1 Gy. 96 h after radiation exposure, *MMP-3* expression was reduced after irradiation with 0.1 and 1.0 Gy, whereas 0.5 Gy did not impact on its expression. For *MMP-13*, expression levels slightly rose, independently of the irradiation dose after 48 h (Figure 2G). However, *MMP-13* expression was reduced 96 h after irradiation with 0.1, 0.5, or 1.0 Gy (Figure 2H). Regarding *OPG* expression, altered expression patterns were only observed after exposure to 1.0 and 2.0 Gy: after 48 h, OPG expression was marginally reduced and after 96 h slightly increased (Figure 2I,J). However, none of these alterations significantly differed from mock-treated controls.

### 2.3. LD-RT Has Divergent Effects on Osteoclast Formation and Function

Osteoclasts are the only cells that are capable of resorbing bone [38]; they are responsible for one of the major problems in RA: painful bone loss that cannot be reversed [47]. This is why a timely treatment is necessary in order to prevent further damage [48]. Furthermore, it is known that higher doses of radiation can have bone-damaging effects [49]. Therefore, it was of interest to examine whether LD-RT has similar effects on healthy osteoclasts as on ones in an inflammatory micro-environment [50]. Thus, differentiation assays were performed in order to assess osteoclast numbers, as well as pit formation assays in order to obtain hints about osteoclast functionality. Additionally, qPCR analyses were performed for prominent osteoclast regulatory molecules.

Bone marrow cells that were isolated from the long bones (tibia and femur) of healthy mice were found to differentiate into significantly and slightly more osteoclasts when being irradiated with 0.1 Gy (*p* = 0.0274) or 0.5 Gy (*p* = 0.6994), respectively. In order to see, whether treatment with LD-RT also leads to altered developmental patterns on a genetic level, we further performed qPCR analysis of the differentiated osteoclasts.

**Figure 3:**
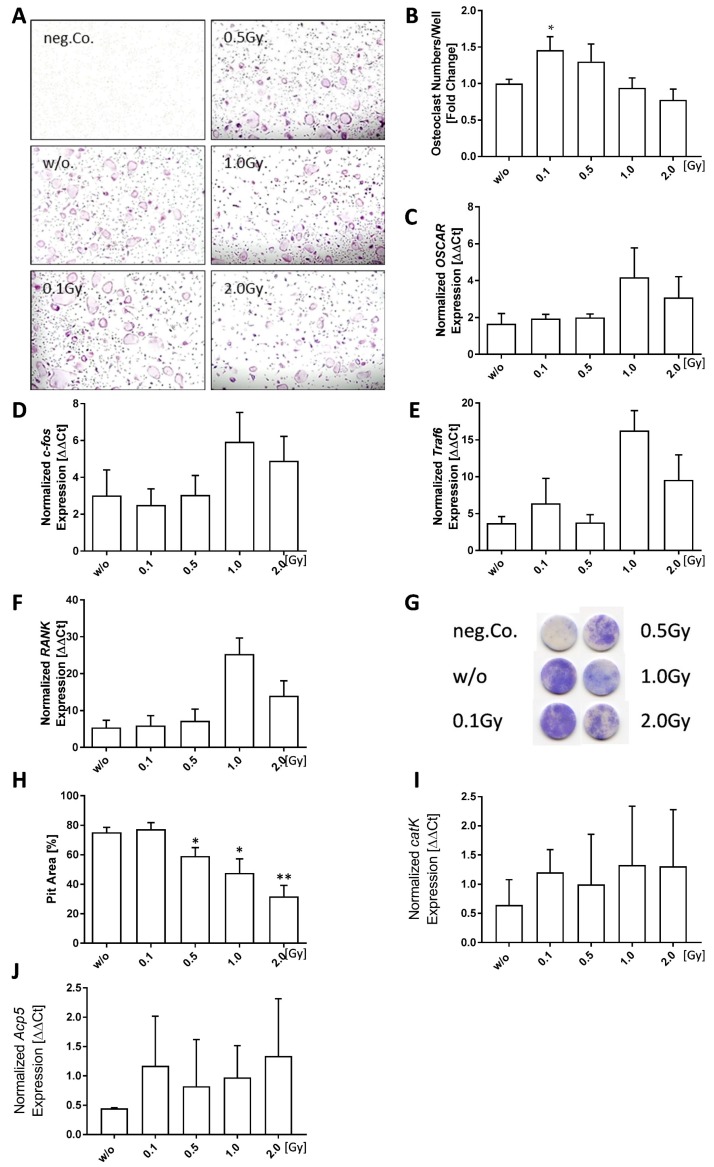
Low doses of ionizing radiation slightly reduces bone resorbing abilities of healthy osteoclasts. Osteoclasts were differentiated from bone-marrow cells from C57Bl/6 animals with 10 ng/mL M-CSF and 50 ng/mL RANK-L over a time period of 6 days (**A**,**B**). Cells were irradiated with various doses of X-rays 24 h after seeding. Differentiated cells were stained for TRAP (**A**) and counted (**B**), whereas TRAP^+^ cells with at least three nuclei were considered to be osteoclasts. RNA extraction was done using the phenol-chloroform method and subsequent quantitative PCR (qPCR) was performed via SYBR green qPCR analyses (**C**–**F**,**I**,**J**). Normalization was done using the housekeeper genes *HPRT* and *HMBS*. Pit formation assay was performed on bone under the same condition as differentiation assays. However, at the end of the incubation period (14 days), bone slices were stained using toluidinblue (TB) (**G**) and pit areas were quantified using ImageJ (**H**). Representative images (**A**) were taken at 5× magnification. Depicted is the data from four (**A**–**F**,**I**,**J**) or two (**G**,**H**) independently performed experiments, each performed in triplicates. Data is presented as mean ± SEM and analysed by two-tailed Mann-Whitney-U test in comparison to untreated controls, with the exception of (**B**) which was tested positive for normal distribution and analysed using student’s *t*-test. (* *p* < 0.05, ** *p* < 0.01).

C-fos is a transcription factor that acts a switch between osteoclast and macrophage differentiation, and it has also been shown, that osteoclasts do not form in its absence [51]. We found *c-fos* expression to be unaffected by LD-RT up to a dose of 0.5 Gy (Figure 3D); however, expression levels were slightly increased after exposure to 1.0 or 2.0 Gy. Similar results were found for the expression of *osteoclast-associated receptor* (*OSCAR*), a regulatory molecule specific to osteoclasts (Figure 3C). As it has been shown before that the addition of soluble OSCAR to osteoblast/osteoclast co-cultures in vitro abrogates the ability of osteoclasts to resorb bone, OSCAR is considered to be an important regulator of functional osteoclast formation that is specific to the bone [52]. The expression of two further factors that are involved in osteoclastogenesis, RANK (Figure 3F) and Traf-6 (Figure 3E), was also slightly increased after 1.0 or 2.0 Gy. During osteoclastogenesis, RANK-L binds its receptor RANK on progenitor cells and initiates intracellular adaptor proteins, such as c-fos and tumor necrosis factor receptor associated factor-6 (Traf-6) [53], ultimately resulting in the fusion of precursor cells and the formation of multinucleated osteoclasts. Among the several adaptor proteins that are involved in the formation of osteoclasts, Traf-6 is one of the most important ones and a lack of it results in unusually high levels of mineralized bone due to a reduced rate of osteoclastogenesis [54].

We subsequently aimed to examine whether enhanced osteoclast numbers also lead to increased bone resorption. For this, pit formation assays on bone slices were performed (Figure 3G,H). In contrast to increased numbers of differentiated osteoclasts, we found significantly reduced pit formation starting at a dose of 0.5 Gy (0.5 Gy: *p* = 0.0221; 1.0 Gy: *p* = 0.0140; 2.0 Gy: *p* = 0.0023). Additionally, we checked for gene expression of lytic enzymes *Acp5* (Figure 3J) and *cathepsin K* (*catk*) (Figure 3I) at the end of the differentiation process (d6). A slight increased gene expression for both enzymes starting already after irradiation with 0.1 Gy was seen.

### 2.4. LD-RT Fosters Bone Formation and Temporarily Upregulates OPG Secretion in Osteoblasts

As bone resorption and bone formation are processes that are linked to and regulate each other [55], we also focused on the effects of LD-RT on osteoblasts. In addition, in vivo studies have already shown that 0.5 Gy of X-rays are able to modulate osteoblast mineralization and promote fracture healing in a model system using rats [56].

**Figure 4:**
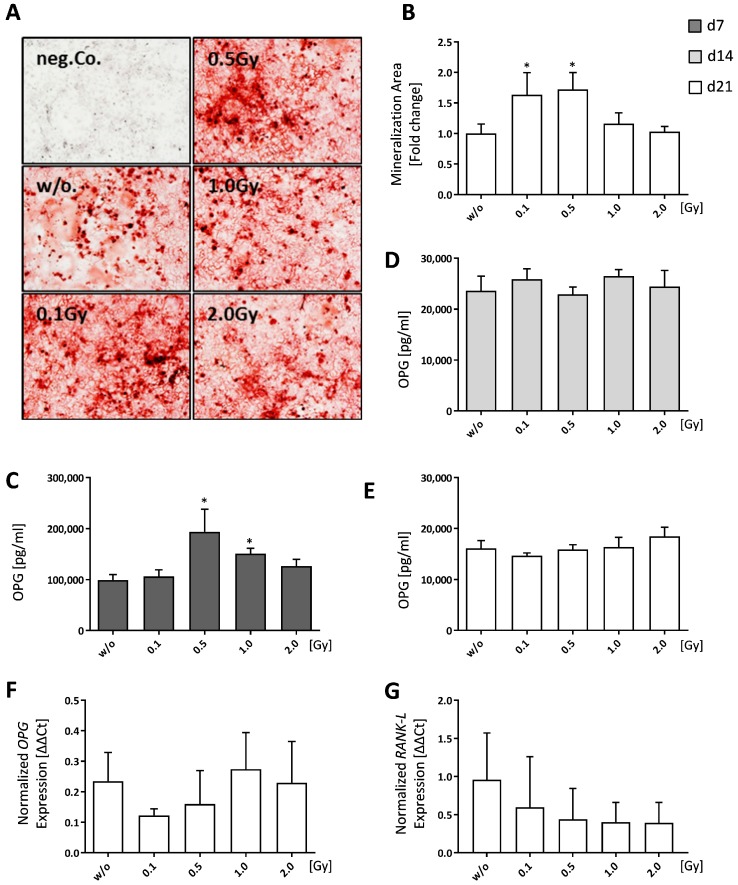
Low doses of ionizing radiation have a positive impact on bone formation of healthy osteoblasts. Pre-osteoblasts were isolated from the calvariae of 3–6 days old neonatal mice and seeded at P2. Cells were allowed to grow until 80–90% confluency before mineralization medium (PromoCell) was added. Irradiation was carried out 24 h after addition of mineralization medium. After 21 days, bone nodules were stained using alizarin red and mineralization area was calculated using ImageJ. (**A**) Shows exemplary alizarin red images taken at 5× magnification. (**B**) Shows mineralization area as assessed using ImageJ. (**C**–**E**) display secreted OPG levels as determined via ELISA and (**F**,**G**) show quantitative PCR (qPCR) data after phenol-chloroform RNA extraction with subsequent gene expression analysis via SYBR green qPCR analyses using housekeepers *B2M*, *ACTB*, *TBP*, and *HMBS* for normalization. Depicted is joint data from two independently performed experiments with three independently generated osteoblast lines, each performed in triplicates. For qPCR analyses cell lysates from triplicates were pooled. Data is presented as mean ± SEM and analysed by two-tailed Mann-Whitney-U test in comparison to untreated controls (* *p* < 0.05).

We observed that calvariae-derived osteoblasts showed significantly increased mineralization after exposure to doses of 0.1 Gy (*p* = 0.0240) or 0.5 Gy (*p* = 0.0151) (Figure 4A,B). OPG release by osteoblasts was increased only at earlier time point (d7) after exposure to 0.5 Gy (*p* = 0.0411) or 1.0 Gy (*p* = 0.0152) (Figure 4C). However, later on (day 14 and day 21), the OPG levels were not significantly altered by radiation exposure (Figure 4D,E).

We further found that, in particular, doses of 0.5 and 1.0 Gy led to a slight reduced expression of *opg* in osteoblasts (Figure 4F), while *RANK-L* expression was slightly decreased in a dose-dependent manner (Figure 4G).

### 2.5. LD-RT Slighlty Increases Cell Death and Alters Immune Cell Populations in Bone-Marrow of C57bl/6 Mice

Since LD-RT does not only influence the cells within the irradiated joints, but also those of the bone-marrow, we also examined cell death rates as well as immune cell composition of bone-marrow following radiation exposure.

**Figure 5:**
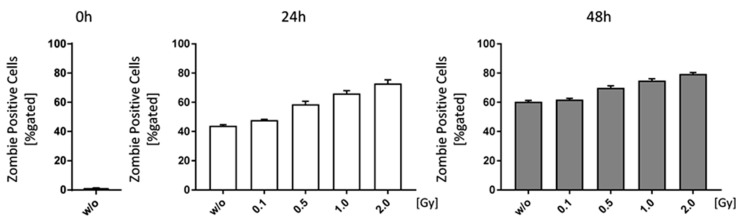
Low doses of ionizing radiation marginally increase cell death rates of bone-marrow. Bone-marrow cells were isolated from the long bones of male C57Bl/6 mice. For analyses 1 × 10^6^ cells were seeded in duplicates and analyzed at different time points (0, 24 and 48 h). In order to distinguish live from dead cells a zombie dye (BioLegend) and multicolor flow cytometry have been used. Zombie negative cells have been considered live, whereas zombie positive cells were counted as dead. Depicted is joint data from one experiment with three 6 weeks old male mice whose bone-marrow has been analyzed independently and in duplicates. Data is presented as mean ± SEM and analysed by two-tailed Mann-Whitney-U test in comparison to untreated controls.

Starting with an irradiation dose of 0.1 Gy, cell death rates in bone marrow cells of C57Bl/6 mice were slightly increased (Figure 5). However, cell death also rose independently of the irradiation (*w*/*o* 0 h: 1.36%; *w*/*o* 24 h: 43.94%; *w*/*o* 96 h: 60.44%), as there were no survival stimuli added to the medium, and in a larger extent than radiation-induced cell death rates (e.g., 2.0 Gy 24 h: 72.85%; 2.0 Gy 48 h 79.45%).

In a next step, we were interested whether radiation exposure has an impact on the composition of immune cells within the bone-marrow.

**Figure 6:**
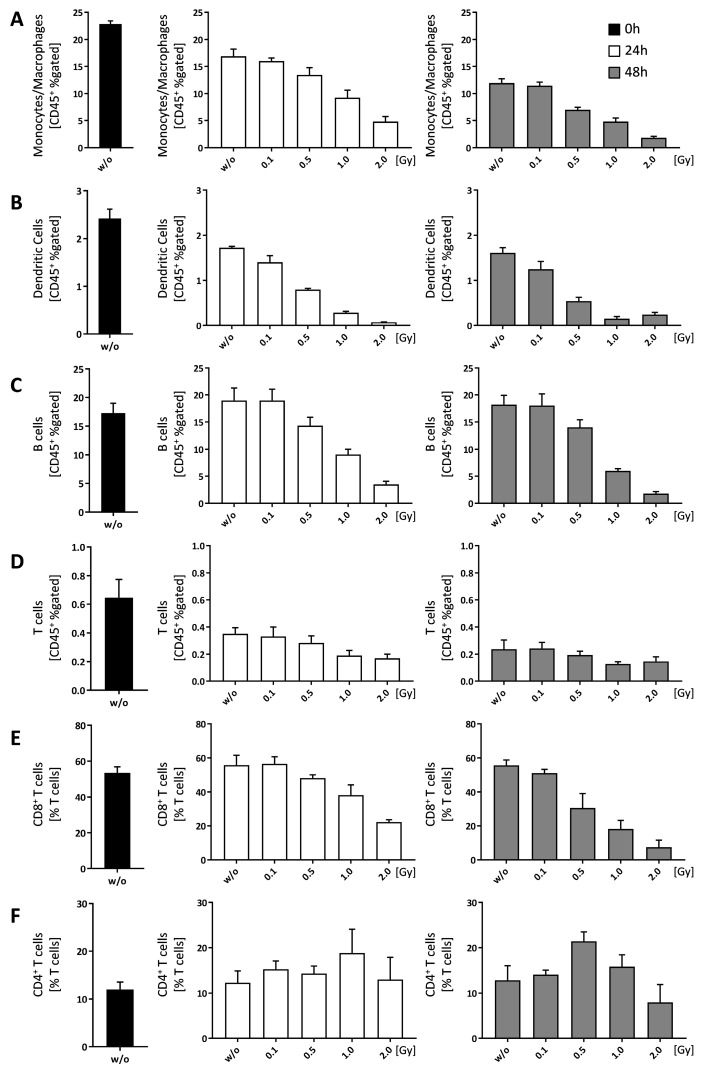
Ionizing radiation alters immune cell composition in bone-marrow of C57Bl/6 mice. Bone-marrow cells were isolated from the long bones of male C57Bl/6 mice. For analyses 1 × 10^6^ cells were seeded in duplicates and analyzed at different time points (0, 24 and 48 h). In order to distinguish live from dead cells a zombie dye (BioLegend, San Diego, CA, USA) was used and the displayed results are all related to living cells. CD45^+^ cells have then been gated into immune cell subpopulations according to specific surface markers: (**A**) Monocytes/Macrophages; (**B**) Dendritic cells; (**C**) B cells; (**D**) T cells; T cells have been further classified into CD8^+^ (**E**) and CD4^+^ (**F**) T cells, respectively. Depicted is joint data from one experiment with three 6 weeks old male mice whose bone-marrow has been analyzed independently and in duplicates. Data is presented as mean ± SEM.

Most of the immune cell populations within the bone-marrow were reduced in a dose-dependent manner, with the exception of CD4^+^ T cells (Figure 6F). While CD8^+^ T cells were reduced in a dose dependent manner (Figure 6E), the numbers of CD4^+^ T cells remained more or less stable with a slight increase at 1.0 Gy (24 h) and 0.5 Gy (48 h), respectively (Figure 6F).

## 3. Discussion

In order to gain insights about the impact of the basal/initial inflammatory state on the anti-inflammatory modes of action of LD-RT and for radiation protection purposes, joint examinations on how radiation modulates the phenotype and function of key cells in joints are needed.

FLS are a vital part of the inflammatory pannus and show tumor like features, such as enhanced resistance to apoptosis and invasive growth [57]. The expansion rate of FLS in RA could further be a result of an imbalanced cell survival and cell death ratio alongside increased proliferation [27,28]. LD-RT has already been shown to induce apoptosis in e.g., PBMCs [17,42]. We thus hypothesized that LD-RT might also induce apoptosis and further reduce the numbers of viable FLS. Even though the number of non-inflammatory, healthy FLS was reduced starting from 0.5 Gy of irradiation, apoptotic cells were only enhanced after a single high dose of 2.0 Gy with no impact on necrosis (Figure 1). These findings highlight that LD-RT has no harmful effects for healthy FLS, and, as we found an increased apoptosis induction only at a higher dose, pleads for secure anti-inflammatory responses in the low-dose range [19].

Further, it is known that RA FLS produce large amounts of inflammatory cytokines, such as IL-6. The latter is not only playing a role in initiating and sustaining inflammation, but also in bone metabolism, as it is known to also increase osteoclastogenesis [54,58]. Therefore, we were interested whether LD-RT also has an impact on cytokine secretion of healthy FLS and revealed that it did not have an influence on IL-6. As LD-RT has been shown to also have an impact on TGF-β secretion of activated cells [17], we further checked for TGF-β release in supernatants of FLS cultures. Only after exposure to a single dose of 0.5 Gy, significant enhanced amounts of this anti-inflammatory cytokine were seen. One could speculate that anti-inflammatory modes of action of LD-RT do not only refer to amelioration of inflammation, but also to induction of a more anti-inflammatory milieu based on healthy FLS.

Further, no significant changes in expression of two adhesion molecules, *VCAM1* and *cdh11*, were observed. However, a biphasic expression pattern of VCAM1 and cdh11 was observed with stable expression levels only after exposure to 0.5 Gy (Figure 2). We therefore conclude that LD-RT with clinically applied 0.5 Gy as dose per fraction has no disadvantages in healthy FLS with regards to adhesive properties and in synovial lining formation [27,44].

A family of destructive molecules secreted by FLS is the MMPs: They are strongly involved in progressive cartilage degradation as MMPs break down proteoglycans [59,60,61]. We found that expression patterns of MMPs vary depending on time and dose: 48 h after radiation exposure, *MMP-3* expression was initially unaffected by LD-RT with the exception of a slight reduction after 0.1 Gy (Figure 2E). However, 96 h post-irradiation, a similar biphasic expression pattern as for *VCAM1* and *cdh11* expression was observed, with no alteration from the untreated levels at especially 0.5 Gy (Figure 2F). In contrast, *MMP-13* expression initially rose slightly in comparison to the control (Figure 2G), however, after 96 h, its expression was slightly reduced in a dose range of 0.1 to 1.0 Gy. This suggests that LD-RT in the low-dose range, and especially at 0.5 Gy, has no negative effect regarding FLS-mediated cartilage destruction. Furthermore, these results also reflect a big challenge in biomolecular radiation research that has strongly to consider that radiation responses are rather dynamic ones and they often follow non-linear dose relationships. In osteoimmunological modifications FLS can further contribute to the bone metabolism through the expression of OPG. No differences in comparison to untreated controls for doses up to 0.5 Gy in its expression were observed in healthy FLS (Figure 2I,J).

A misbalanced osteoclast activity is one of the hallmarks of RA [30,55]. It is also known that bone loss is a possible feature in high-dose radiotherapy and increased osteoclast numbers and activity have been shown after 2.0 Gy whole body irradiation in mice [62]. We therefore investigated the effects of LD-RT on the differentiation process of osteoclasts and found a significant increase in differentiated osteoclasts after exposure to 0.1 Gy, as well as a slight increase after 0.5 Gy (Figure 3B). However, functional testing of the ability of these osteoclasts to resorb bone showed significantly reduced resorption areas at a dose range from 0.5 to 2.0 Gy (Figure 3H), which possibly counteracts increased osteoclast numbers. Expression patterns of genes playing a role in osteoclast development, such as *c-fos*, *OSCAR*, *RANK*, and *Traf6* were unaffected by LD-RT with single doses up to 0.5 Gy (Figure 3C–F). Doses of 1.0 and 2.0 Gy, however, resulted in an increase with peak expression at 1.0 Gy. The expression of the lytic enzymes *catK* and *Acp5* varied and was only slightly increased after radiation in comparison to the control. Nevertheless, once again, 0.5 Gy exposure resulted in similar expression patterns as those of untreated controls.

Bone resorption and built up is usually a tightly linked process that also closely connects osteoclasts and osteoblasts with each other, as osteoblasts are able to influence osteoclastogenesis [55,63]. Chen et al. found an increased bone formation after LD-RT [56]. Our results confirm that irradiation of pre-osteoblasts with 0.1 or 0.5 Gy increases the mineralized area, thus indicating a positive effect on bone formation (Figure 4B). However, as we also detected increased osteoclast numbers in the same dose range (Figure 3B), this LD-RT-mediated effect on osteoblasts possibly counteracts increased osteoclast numbers in the healthy situation. Additionally, we monitored OPG secretion over the entire mineralization process and found that levels were especially enhanced within the first seven days following irradiation (Figure 4C). This points towards a short-term inhibition of osteoclast formation, as OPG is a well-known negative regulator of osteoclastogenesis [64,65,66]. Gene expression analyses via qPCR on d21 revealed a biphasic expression pattern of *OPG* after irradiation with low, intermediate and higher doses with an expression minimum at 0.1 Gy and a maximum at 1.0 Gy (Figure 4F). In contrast, *RANK-L* expression showed a dose-dependent decrease (Figure 4G), suggesting a possible reduced induction of osteoclast formation after LD-RT.

Finally, we aimed to investigate how LD-RT influences immune cell composition in the bone marrow. First we checked for cell death induction and found a slight, but not significant impact of LD-RT on the normal fast death rates of bone marrow cells when examined ex vivo (Figure 5). When looking at immune cell composition, we found that, starting with a dose of 0.5 Gy, monocytes/macrophages, DCs, B cells, and CD8^+^ T cells were reduced, but CD4^+^ T cells increased after 1.0 Gy (24 h) and 0.5 Gy (48 h) (Figure 6). As there were no significant alterations in total cell numbers, this might point to the fact that LD-RT does not necessarily lead to immune cell death, but mainly rather a shift in sub populations, as seen in the T cell subsets. Rühle et al. found a similar outcome in the blood of patients that received radon spa therapy [25].

One can conclude that LD-RT, in particular with the clinically relevant single dose per fraction of 0.5 Gy, has no harmful effects on key cells of healthy non-inflamed joints, namely on FLS, osteoclasts, osteoblasts, and immune cells. If alterations occur they rather point towards anti-inflammation. One has to stress that many of the observed effects followed a discontinuous dose-dependency and were dynamic ones regarding time after radiation exposure. These facts, together with the dependence of radiation responses on the initial inflammatory state, highlight the big challenges of biomolecular radiation research of the future, as we nowadays know that cells of the immune system and bone metabolism react differently when being inflamed or non-inflamed/healthy [50].

## 4. Materials and Methods

### 4.1. Mouse Keeping

C57Bl/6 mice were maintained in a SPF facility under sterile atmosphere at the animal facility of the Universitätsklinikum Erlangen (Franz-Penzoldt-Center). The animal procedures have been approved by the “Regierung of Unterfranken” (Approval Numbers: 55.2-DMS-2532-2-114 from 13 November 2015 and 10 December 2015; TS-3/14 from 21 February 2014; TS 5/2016 from 16 April 2016) and they were conducted in accordance with the guidelines of Federation of European Laboratory Animal Science Associations (FELASA).

### 4.2. Ex Vivo Irradiation Procedure

Irradiation of ex vivo cells cultures was carried out using an Isovolt Titan X-ray tube (120 kV, 22.7 mA, variable time, GE Inspection Technologies, Hürth, Germany) equipped with a 0.5 mm copper filter and a 5 cm Plexiglas^®^ plate. Unless stated otherwise, cells were irradiated 24 h after plating with a single dose of X-rays (0.1, 0.5, 1.0, and 2.0 Gy).

### 4.3. Fibroblast-Like Synoviocytes (FLS) Cultures

FLS cultures were prepared in accordance with a protocol provided by Armaka et al. [67]. Briefly, hind paws of C57Bl/6 mice were skinned and incubated for 4 h in a 1% collagenase IV type solution (Gibco, Carlsbad, CA, USA) at 37 °C with shaking at 1400 rpm. Cells then were pooled and kept at standard culture conditions (37 °C, 5% CO_2_; 95% humidity) in a medium containing 50% Dulbecco’s modified Eagle medium (DMEM; Pan Biotech, Aidenbach, Germany) and 50% F-12 medium (Gibco) supplemented with 10% fetal bovine serum (FBS; Biochrom, Berlin, Germany), 1% penicillin/streptomycin (PS; Gibco), and 1% Low serum growth supplement (LSGS; Gibco). Cell purity was analyzed using flow cytometry, whereas, CD11b^−^, CD54^+^, and CD106^+^ cells were considered to be FLS. In total, three independent FLS pools at P5 were used. Experiments were carried out by seeding 25,000 cells in triplicates in 6-well plates for the evaluation of cell growth, RNA isolation, and ELISA analysis or in T25 cell culture flasks for cell death analysis. 48 and 96 h after irradiation, respectively, samples were taken and processed.

### 4.4. Scratch Assay

In order to measure cell migration in vitro [68], we used a scratch assay. For this, FLS were seeded at a density of 25,000 cells/well in 6-well plates each brought out in triplicates, and cultivated at standard conditions until they had reached 100% confluency. 12 h prior to administering the scratch area, cells were irradiated using various doses of X-rays. Images then were captured regularly. For each scratch, three images/well were taken at pre-marked spots at 40× magnification using a Zeiss PrimoVert microscope equipped with an AxioCam ERc5s (Zeiss, Carl Zeiss Ag, Oberkochen, Germany). Scratch areas were evaluated using the TScratch Software (V1) (CSE Lab, Zürich; Switzerland). Scratch areas of the three images/well were calculated and their means were used for further analysis.

### 4.5. Matrigel Invasion Assay

0.1 × 10^5^ FLS were seeded into Geltrex (Thermo Fisher Scientific, Waltham, MA, USA) coated Transwells^®^ (Sigma-Aldrich, Darmstadt, Germany) in medium *w*/*o* additives and normal medium was placed into the surrounding wells. 6 h after seeding cells were irradiated and incubated for 3 days at standard culture conditions. At the end of the incubation periode, cells on the outside of the transwell were fixed with 2% glutaraldehyde/PBS and stained with 1% crystal violet. Stained transwells then were scanned using an Epson Perfection 500 V Photo scanner (Epson, Suwa, Japan) and analyzed using ImageJ.

### 4.6. Osteoclast Culture

Bone marrow-derived osteoclasts (OCs) were isolated from the long bones of C57Bl/6 mice and kept in modified Eagle’s medium type α (α-MEM; Gibco) supplemented with 10% FBS (Sigma-Aldrich, Darmstadt, Germany), 1% PS (Gibco) at standard culture conditions (37 °C, 5% CO_2_, 95% humidity) overnight. Differentiation was then carried out by adding 10 ng/mL M-CSF (PeproTech, Rocky Hill, NJ, USA) and 50 ng/mL RANK-L (PeproTech) to the culture medium. Cells were seeded at a density of 1Mio cells/Well in 24-well plates for RNA analysis and 500,000 cells/well in 48-well plates for TRAP stain, respectively. OC differentiation was evaluated via TRAP stain using a leukocyte acid phosphatase kit (Sigma-Aldrich), according to the manufacturer’s instructions. TRAP positive cells with three or more nuclei were counted as osteoclasts.

### 4.7. Pit Formation Assay

In order to determine osteoclast functionality, bone marrow cells were seeded onto cortical bovine bone slices (boneslices.com) in a 96-Well format at a density of 1 Mio cells/Well and cultured for 14 days in the presence of M-CSF (10 ng/mL) and RANK-L (50 ng/mL). 24 h after seeding, cells were irradiated, as described above. At the end of the cultivation process, cells were detached from bone slices using 0.25 M ammonium hydroxide for 5 min, followed by washing in PBS and staining using 1% TB. Bone slices then were scanned using an Epson Perfection 500 V Photo scanner and analyzed using ImageJ.

### 4.8. Osteoblast Culture

For osteoblast cultures mesenchymal cells were isolated from the calvariae of 3–6 days old neonatal mice. In brief, calvariae were digested in α-MEM medium (PAN Biotech, Aidenbach, Germany) supplemented with 0.1% collagenase type IA (Sigma-Aldrich) and 0.2% dispase II (Roche, Basel, Switzerland) at 37 °C on a shaker for 5 × 10 min. Fractions 2–5 were collected and cells were cultured and expanded until P2 to P3. At subconfluency state, cells were plated at 1 × 10^4^ cells/cm^2^. Mineralization assays were carried out in 12-well plates by changing the medium to osteoblast mineralization medium (PromoCell, Heidelberg, Germany) at 100% confluency and cells were irradiated 24 h after the first medium change. Mineralization media was used according to the manufacturer’s recommendation and formation of bone nodules was evaluated at day 21 using Alizarin red stain (Millipore, Darmstadt, Germany). For analyses, total wells were scanned and images then were analyzed using ImageJ software (Version 1.46r).

### 4.9. Bone Marrow Culture

Bone marrow cells were isolated from the long bones of C57Bl/6 mice, erythrocytes were lysed, and the remaining cells were seeded at a density of 1 × 10^6^ cells in duplicates. Immune cell composition was analyzed via flow cytometry at different time points (0, 24 and 48 h). Cells were seeded into six-well plates in modified Eagle’s medium type α (α-MEM; Gibco) supplemented with 10% FBS (Sigma-Aldrich, Darmstadt, Germany), 1% PS (Gibco) without any additional surviving factors at standard culture conditions (37 °C, 5% CO_2_, 95% humidity). Cells were then irradiated and cultured for 24 and 48 h, respectively, before analyzation.

### 4.10. Enzyme Linked Immunosorbent Assay (ELISA)

For further analyses, supernatants of cell cultures were taken at the respective time points, and, in the case of FLS, levels of IL-6 (Biolegend, San Diego, CA, USA) and TGF-beta (eBioscience, San Diego, CA, USA) were measured according to the manufacturer’s instructions. Here, samples were diluted 1:5 prior to the measurement. Supernatants of osteoblast cultures were taken accordingly, diluted 1:100, and OPG levels were determined using an R&D ELISA according to the manufacturer’s instructions.

### 4.11. RNA and Quantitative PCR

Total RNA from respective cell cultures was isolated using TriFast (peqlab, Darmstadt, Germany) and Phenol-chloroform extraction. 0.8 µg of total RNA was transcribed into cDNA using the QuantiTect^®^ reverse transcription kit by Quiagen (Hilden, Germany) according to the manufacturer’s recommendations. Quantitative real-time PCR was carried out using SYBR Green (Thermo Scientific, Waltham, MA, USA) as fluorescent dye. Genes of interest were normalized to at least two housekeepers (see Table 1). Target stability values for these housekeepers was tested and a coefficient variance of <0.5 was accepted as stable. Obtained data was analyzed using the CFX Manager 3.1 software (BioRad, Hercules, CA, USA). Primers were either designed using Primer Blast (NCBI) and manufactured by MWG (Eurofins Genomics GmbH, Ebersfeld, Germany) or by using BioRad PrimePCR products (Bio-Rad Laboratories, Inc., Hercules, CA, USA), respectively. Efficiency of primers was either provided by the manufacturer or determined prior to use.

### 4.12. Flow Cytometry Analyses

For flow cytometry analyses, cells were trypsinated and, in the case of cell death analysis, resuspended in ringer’s solution (Braun, Melsungen, Germany) containing 0.2 µg/mL AnnexinV-FITC (AxV) and 0.4 µg/mL propidiumiodide (PI), and consecutively stained for 30 min at 4 °C in the dark. Cells then were measured with an EPICS XL-MCL (Beckman Coulter, Brea, CA, USA) flow cytometer and evaluated with the Kaluza Analysis software (Beckman Coulter). AxV^−^/PI^−^ cells were considered as live cells, while AxV^+^/PI^−^ and AxV^+^/PI^+^ cells were considered to be apoptotic and necrotic, respectively. In the case of surface staining of FLS, 1 × 10^6^ cells were resuspended in 100 µL 2% FBS/PBS and incubated at 4 °C with saturated fluorochrome-labeled antibodies (BD Bioscience, Franklin Lakes, NJ, USA and eBiosciences, Frankfurt, France) for 30 min in the dark at 4 °C. Measurements were carried out with a Gallios flow cytometer (Beckman Coulter). For phenotyping of FLS cultures, the following antibodies were used: CD11b-FITC (BD #557396), CD54-PE (eBioscience # 12-0541), CD90.2-PE (eBioscience #12-0903), and CD106-FITC (eBioscience #11-1061). Evaluation of the samples was done with the Kaluza Analysis software.

For immune phenotyping, 1 × 10^6^ cells were stained using the following antibodies and stained for 30 min at 4 °C in the dark: Zombie NIR (BioLegend, San Diego, CA, USA); MHC-II-eFluor660 (eBioscience #48-5321); Ly-6G-PE-Cy7 (BD #560601); Ly-6C-FITC (BD #553104); CD11b-APC (eBioscience #17-0112); Siglec-F-PE (BD #552126); CD45.2-PerCP-Cy5.5 (eBioscience #45-0454); PDCA-1-BV650 (Biolegend #127019); CD11c-BV510 (BD #562949); CD62L-PE-Cy7 (BD #56516); CD8-BV605 (BioLegend #100744); FcεR1α-PE (BioLegend #134308); Zombie Aqua (BioLegend); CD4-FITC (BD #553729); CD19-APC-Cy7 (BD #561737); CD3-V450 (BD #560801); CD49b-APC (BioLegend #108910); and, PD-1-PE-Dazzle594 (BioLegend #109116).

### 4.13. Statistical Analysis

Statistical analysis was performed with GraphPad Prism software (GraphPad software, Inc., San Diego, CA, USA). All data is presented as mean ± SEM and tested for normal distribution and variance equality. Ultimately, the non-parametric two-tailed Mann-Whitney-U test was used for analyzation in comparison to mock-treated controls. Significances are indicated as follows: * *p* < 0.05, ** *p* < 0.01, *** *p* < 0.001.

## Figures and Tables

**Table 1:** List of used Primers and their respective sequences in alphabetic order.

MWG Primers		
Symbol	Forward [5′→3′]	Reverse [3′→5′]
*Acp5*	CGACAAGAGGTTCCAGGAGA	TTCCAGCCAGCACATACCAG
*ACTB*	ACAGCTTCTTTGCAGCTCCTTCG	ATCGTCATCCATGGCGAACTGG
*B2M*	CTGCTACGTAACACAGTTCCACCC	CATGATGCTTGATCACATGTCTCG
*catK*	GGC CAG TGT GGT TCC TGT T	CAG TGG TCA TAT AGC CGC CTC
*cdh11*	TTTCCACAGAGCGTGTACCA	GGGTCTTTAGCCTTCACCCTT
*c-fos*	CGACCATGATGTTCTCGGGT	TCGGCTGGGGAATGGTAGTA
*GAPDH*	AGGTCGGTGTGAACGGATTTG	GGGGTCGTTGATGGCAACA
*HMBS*	GAGTCTAGATGGCTCAGATAGCATGC	CCTACAGACCAGTTAGCGCACATC
*HPRT*	GTTGGGCTTACCTCACTGCTTTC	CCTGGTTCATCATCGCTAATCACG
*MMP-3*	GCTGTCTTTGAAGCATTTGGGTT	ACAATTAAACCAGCTATTGCTCTTC
*OSCAR*	TGCCTGGAGATGGACAGAGA	GCCTGACAGTGTGGTGAAGA
*Tnfrsf11a*/*RANK*	GCC CAG TCT CAT CGT TCT GC	GCA AGC ATC ATT GAC CCA ATT C
*Tnfsf11*/*RANK-L*	ACC AGC ATC AAA ATC CCA AG	TTT GAA AGC CCC AAA GTA CG
*TBP*	TCTGAGAGCTCTGGAATTGTACCG	TGATGACTGCAGCAAATCGCTTG
*Traf6*	AAA GCG AGA GAT TCT TTC CCT G	ACT GGG GAC AAT TCA CTA GAG C
*VCAM-1*	CCCACCATTGAAGATACCGGG	GGGGGCAACGTTGACATAAAG
**BioRad primers**		
**Symbol**	**Unique Assay ID**	
*MMP-13*	qMmuCID0025884	
*RPS18*	qMmuCED0045430	
*Tnfrsf11b*/*OPG*	qMmuCID0027158	

## Abbreviations

ACTB	β-actin
Acp5	acid phosphatase 5, tartrate resistant
AxV	Annexinv-FITC
CD	Cluster of differentiation
cdh11	Cadherin 11
d	day
ELISA	Enzyme linked immunosorbent assay
FLS	Fibroblast-like synoviocytes
GAPDH	Glyceraldehyde-3-phosphate dehydrogenase
Gy	Gray
ICAM1	Intercellular adhesion molecule 1
IL	Interleukin
iNOS	Inducible nitric oxide synthase
LD-RT	Low dose-radiotherapy
M-CSF	Macrophage-colony stimulating factor
MMP	Matrixmetalloproteinase
NO	Nitric oxide
OA	Osteoarthritis
OC	Osteoclast
OPG	Osteoprotegerin
OSCAR	osteoclast-associated receptor
PBMC	Peripheral blood mononuclear cells
PI	Propidiumiodide
RA	Rheumatoid arthritis
RANK-L	Receptor activator of nuklear factor κ B ligand
RPS18	Ribosomal protein 18
RT	Radiation therapy
TNF-α	Tumornecrosis factor-α
Traf-6	Tumor necrosis factor associated receptor-6
TRAP	tartrate-resistant acid phosphatase
VCAM1	Vascular adhesion molecule 1
